# Prevalence and predictors for unintended pregnancy among HIV-infected pregnant women in Lira, Northern Uganda: a cross-sectional study

**DOI:** 10.1038/s41598-020-73490-6

**Published:** 2020-10-01

**Authors:** Agnes Napyo, Victoria Nankabirwa, David Mukunya, Josephine Tumuhamye, Grace Ndeezi, Anna Agnes Ojok Arach, Beatrice Odongkara, Paul Waako, Thorkild Tylleskär, James K. Tumwine

**Affiliations:** 1 Department of Public Health, Faculty of Health Sciences, Busitema University, Mbale, Uganda; 2 Department of Paediatrics and Child Health, College of Health Sciences, Makerere University, Kampala, Uganda; 3 School of Public Health, College of Health Sciences, Makerere University, Kampala, Uganda; 4 Centre for International Health, University of Bergen, Bergen, Norway; 5 Department of Nursing and Midwifery, Lira University, Lira, Uganda; 6 Department of Paediatrics and Child Health, Gulu University, Gulu, Uganda; 7 Department of Pharmacology, Faculty of Health Sciences, Busitema University, Mbale, Uganda

**Keywords:** Medical research, Epidemiology, Epidemiology, Public health

## Abstract

Prevention of unintended pregnancies is a global strategy to eliminate mother-to-child transmission of HIV. Factors surrounding unintended pregnancy among women living with HIV are not well understood. We aimed to determine the prevalence and predictors for unintended pregnancy among these women in Northern Uganda. We conducted a cross-sectional survey among 518 women using a structured questionnaire. We asked questions on socio-demographic, reproductive-related and HIV-related characteristics. We conducted multivariable logistic regression and reported adjusted odds ratios. The prevalence of unintended pregnancy was 41.1%. The predictors for unintended pregnancy were: being single (not living with a partner or being in a marital union), having five or more children and taking antiretroviral drugs for long periods of time. HIV counselling services should target women living with HIV who are not in a marital union, those having a higher parity and those who have taken ART for longer periods.

## Introduction

The Human Immunodeficiency Virus (HIV) prevalence in Uganda was 6.0% in 2017^1^. There has been a scale up of prevention of mother-to-child transmission of HIV-1 (PMTCT) services covering over 95% of pregnant women and as a result there has been a significant reduction in the mother-to-child transmission of HIV-1 (MTCT) rate to less than 5%^1^. The global strategy by the World Health Organisation (WHO) for PMTCT is multi-pronged and includes: (a) primary prevention of HIV infection among women of child-bearing age, (b) prevention of unwanted pregnancies among women living with HIV (WLH), (c) prevention of HIV transmission from WLH to their infants and (d) provision of appropriate treatment to WLH and their children^2^. Uganda has taken strides in PMTCT largely because of infections prevented due to the provision of antiretroviral therapy (ART) to pregnant WLH; these strides do not reflect infections averted due to preventing unintended pregnancies^1^. Fertility desires for WLH have increased overtime^3–7^ and so it is eminent to support them in their fertility choices through family planning and contraception. To ensure provision of contraception, the consolidated guidelines for prevention and treatment of HIV in Uganda promote the availability of accessible and comprehensive contraceptive services for WLH to not only meet their birth control needs but to also reduce rates of unintended pregnancy^2^. Prevention of unintended pregnancy through reliable contraceptive methods reduces MTCTof HIV, improves women’s health as well as reduce both maternal and infant mortality among WLH and their offspring^6^. Preventing unintended pregnancy also offers several additional benefits for WLH and their babies by reducing the number of infants who acquire HIV and consequently those who need HIV services as well as increased survival for HIV exposed infants^2^.

Conversely, unintended pregnancy poses health consequences for mothers, their babies and families^8,9^. A woman who conceives and gets pregnant when she does not desire to will not to seek prenatal care early enough^8,10^. A baby born to a mother who did not desire her pregnancy is likely to have low birth weight and other life threatening issues^11^. Both parents to the baby may suffer economic hardship^11^. Most importantly, WLH that experience unintended pregnancy are more likely not to adhere to the ART and hence frequently have elevated viral load counts^12,13^. Such grave consequences undoubtedly increase the risk of MTCT of HIV.

Much as integration of family planning into HIV services exists, the rates of unmet need for contraception remain elevated^6^ ranging from 36 to 75%^14–20^ which results into high rates of unintended pregnancy^19,21^ in sub-Saharan Africa that range from 35 to 71%^3,7,13,17,19,20,22–27^. Unintended pregnancy can also result from incorrect or inconsistent use of contraception^5,24^, interaction of hormonal methods with ART making them less effective^28,29^, miscalculation of safe days while relying on non-modern contraception^19,30^ and contraceptive failure^7,20^.

Existing evidence demonstrates that various factors have been associated with unintended pregnancy among WLH and these are younger age, being single, ethnicity, education status, higher parity, having had a previous abortion, late or no antenatal care attendance, incorrect or inconsistent use of barrier methods, elevated viral load, peri-partum CD4+ immune suppression and long term ART use^5,12,13,22–24,30–32^.

Studies that have been done on this subject matter have mainly looked into predictors for contraceptive use in the context of HIV and only, in a few cases, mentioned the rates of unintended pregnancy but rarely, its predictors. Furthermore, the unmet need for contraception is higher among WLH than their HIV negative counterparts^21^ translating into WLH facing higher rates of unintended pregnancy than their HIV negative counterparts^31^. Factors surrounding unintended pregnancy among WLH in Uganda are still not well understood yet they remain a public health challenge. We therefore aimed to determine the prevalence and predictors for unintended pregnancy among HIV infected pregnant women in Lira, Northern Uganda.

## Methods

### Study design

We conducted a cross-sectional study among HIV infected pregnant women between August 2018 and July, 2019. The exposures of interest were potential predictors which included socio-demographic, reproductive-related and HIV-related factors. The outcome of interest was unintended pregnancy.

### Sample size estimation

We calculated a sample size for detecting a difference between two independent proportions using Stata version 14.0 (StataCorp; College Station, TX, USA). We utilized the statistics, power and sample-size functions. Using the population parameter method with the test of comparing two independent means (0.576 vs. 0.315), we assumed 80% power, 95% confidence interval (CI) and 5% precision. We also assumed that 57.6% of WLH were not in a marital union^33^ and that 31.5% of HIV infected women were married^24^. On running this calculation in the statistical software, we arrived at a sample size of 464. We adjusted the sample size to 516 after accounting for a 10% non-response. We however, included 518 HIV positive pregnant women who were receiving antenatal care at Lira Regional Referral Hospital (LRRH).

### Setting

LRRH serves all the 8 districts of the Lango subregion in Northern Uganda. It is a government-owned health facility at tertiary level that offers health services including maternal and child health services like HIV care, antenatal care and delivery. These services are at no cost to the patients.

LRRH also has an annual outpatient attendance of almost 100,000 patients, annual antenatal care attendance of about 5,000 women and conducts approximately 6–7,000 deliveries annually.

### Participants

HIV infected women were identified, consented and recruited consecutively through the existing Ugandan HIV treatment, care and support program for pregnant women at the PMTCT clinic located within LRRH. Women were eligible for participation if they were: 20 weeks pregnant or more, newly tested for HIV or already established in ART care. Those who were severely ill at the time of recruitment were excluded from the study and referred to appropriate care services.

### Surveys and measures

The interviews were conducted in *Lango* (the main language spoken in the study setting) or English by trained study staff. Interviews consisted of socio-demographic related, reproductive-related and HIV-related information. All measures were translated into *Lango* and back-translated into English to ensure accuracy and minimise interpretation bias. All procedures of the study were performed in accordance with the guidelines and regulations pertaining to all relevant approving bodies.

Unintended pregnancy, the main outcome of the study, was defined in any of the following ways: a pregnancy that occurred when no more children were desired or one that occurred earlier than it was desired or one that occurred when the woman did not desire to become pregnant. We adapted questions from the London Measure of Unplanned Pregnancy, a psychometrically validated measure of the degree of intention of a current or recent pregnancy. Women were asked if the pregnancy came ‘earlier than expected’, ‘later than expected’, ‘when expected’ or ‘not desired at all’. Women who had their pregnancy at the ‘time desired’ or ‘later than expected’ were combined, labelled as the ‘intended’ category and coded 0. Women with an ‘earlier than desired’ or ‘unwanted pregnancy’ were combined into a single group, labelled “unintended pregnancy” and coded 1. Contraceptive use, was defined as any contraceptive method used in the 6 months preceding the pregnancy at the time. Unmet need for contraception was defined as those women who experienced unintended pregnancy and did not use any form of contraception 6 months prior to the pregnancy. Marital status was categorised into married and single. Those who were married or cohabiting were combined into one group, labelled “married” and coded 1. Those who were separated, divorced, widowed or not married were combined into one group, labelled “single” and coded 2. Women who had been pregnant for four or less times including miscarriages, abortions and the pregnancy at the time of the interview were collectively categorised and coded 1 for the variable “parity”, else were coded 2. Duration on treatment was categorised as “< 6 months”, “7–30 months”, “31–119 months” and “≥ 120 months”. Duration on ART of ≥ 120 months (10 years) was referred to as long-term ART use^34,35^ for comparability purposes.

We created a composite index of wealth (socio-economic status) using principle component analysis (PCA). Because the PCA technique allows combination and ranking of a number of variables into smaller and fewer variables without prejudgment, it is considered a more accurate indicator of socioeconomic status than single items such as occupation or possession of particular items^36^. We used PCA on house ownership, availability of electricity in the house, source of drinking water and fuel used for cooking. Scores were obtained and categorized into four groups (quartiles) ranging from the poorest to the least poor.

### Data management and statistical analyses

Data were entered into EpiData software (www.epidata.dk, version 4.4.3.1) by two independent data entrants and exported for analysis into Stata version 14.0 (StataCorp, College Station, Texas, U.S.A.). Continuous data, if normally distributed, was summarised into means and standard deviations and if skewed, was summarised into medians with their corresponding interquartile ranges. Categorical variables were summarised into frequencies and percentages. The proportion of HIV infected women with unintended pregnancies was estimated and its confidence limits calculated using the exact method. We used multivariable generalized linear model regression analysis with a logit link to estimate the adjusted odds ratios of the independent variables on unintended pregnancy while controlling for confounding. All variables with *p* < 0.25 at the bivariate level were included in the initial model at the multivariate analysis. All variables with *p* < 0.1 and those of biological or epidemiologic plausibility (from previous studies) were included in the second model. We checked for confounding by calculating the percentage change in each effect measure by removing or introducing one variable at a time into the second model. If a variable caused more than 10% change in any effect measure, then it was considered a confounder. Using the Likelihood-ratio test, we found that the first and second models were not significantly different. Therefore we adopted the second model as our final model. We used the visual inspection factor to check for collinearity among all the variables that were included in the initial model.

### Ethical considerations

Approval to conduct the study was granted by the Makerere University School of Medicine Research and Ethics Committee, the Norwegian Regional Committee for Medical and Health Research Ethics in the West, and the Uganda National Council for Science and Technology. Meetings were held with the Lira district health officer and LRRH director to grant administrative clearance to conduct the study. Additional meetings were held with the counsellors who work within the PMTCT clinic to introduce to them the study and its procedures and to request them to identify, mobilise and link willing participants with the research team. All participants provided written informed consent confirming their voluntary participation in the study. Those that declined participation were not penalised or denied standard health care. Confidentiality and privacy of all data collected was observed during the course of the study through restricted access.

## Results

**Figure 1:**
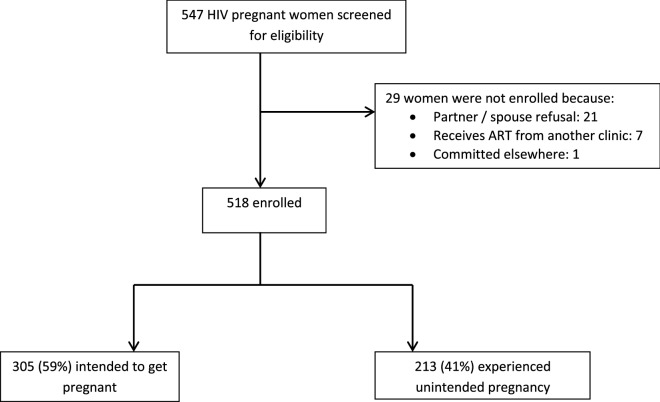
Flow diagram illustrating rationale for screening, enrolment and non-enrolment.

A total of 547 HIV infected pregnant women were screened for eligibility from the antenatal care clinic at Lira Regional Referral Hospital (LRRH) (Fig. 1). A total of 518 women participated in the study. Women did not participate in the study if their partner declined their participation, if they received ART care from another facility other than LRRH or if they were committed elsewhere. The participants had a mean age of 29.2 (SD 5.5). Two hundred and fifty (48.3%) had attained a formal education for a duration of at least seven years or more. The majority of the women were married (or cohabiting) and not formally employed. They were predominantly Christian and *Lango* speaking (Table 1). Most of the women had been pregnant for at least four times including the pregnancy at the time of the study with more than half having a pregnancy ranging from 20 to 28 weeks of gestation. A considerable proportion of these women had disclosed their HIV status and most had disclosed to their spouse. Almost half of the participants reported having used an effective form of contraception (oral contraceptives, intrauterine devices, injectable contraceptives or implants) six months prior to the pregnancy at the time of the study (Table 2).

**Table 1:** Baseline demographic characteristics of HIV infected pregnant women receiving antenatal care at Lira Regional Referral Hospital.

Characteristics	Total	Pregnancy intended	Pregnancy not intended
	(N = 518)	(N = 305)	(N = 213)
	n (%)	n (%)	n (%)
*Socio-demographic*			
**Age**			
≤ 20 years	35 (6.8)	23 (7.5)	12 (5.6)
21–29 years	229(44.2)	150 (49.2)	79 (37.1)
≥ 30 years	254 (49.0)	132 (43.3)	122 (57.3)
**Education**			
0–6 years	268 (51.7)	143 (46.9)	125 (58.7)
7–10 years	171 (33)	105 (34.4)	66 (31)
11–13 years	51 (9.9)	35 (11.5)	16 (7.5)
≥ 14 years	28 (5.4)	22 (7.2)	6 (2.8)
**Marital status**			
Married	484 (93.4)	295 (96.7)	189 (88.7)
Single	34 (6.6)	10 (3.3)	24 (11.3)
**Employment status**			
Employed	207 (40)	129 (42.3)	78 (36.6)
Not employed	311 (60)	176 (57.7)	135 (63.4)
**Religious affiliation**			
Christian	500 (96.5)	292 (95.7)	208 (97.7)
Moslem	18 (3.5)	13 (4.3)	5 (2.3)
**Ethnic belonging**			
Langi	472 (91.1)	275 (90.2)	197 (92.5)
Other	46 (8.9)	30 (9.8)	16 (7.5)
**Socioeconomic index**			
Group 1 (poorest)	139 (26.8)	80 (26.2)	59 (27.7)
Group 2	122 (23.6)	69 (22.6)	53 (24.9)
Group 3	170 (32.8)	96 (31.5)	74 (34.7)
Group 4 (least poor)	87 (16.8)	60 (19.7)	27 (12.7)

**Table 2:** Other characteristics.

Characteristics	Total	Pregnancy intended	Pregnancy not intended
	(N = 518)	(N = 305)	(N = 213)
	n (%)	n (%)	n (%)
*Reproductive-related*			
**Parity**			
≤ 4	375 (72.4)	247 (81.0)	128 (60)
≥ 5	143 (27.6)	58 (19.0)	85 (40)
**Gestational age (in weeks)**			
20–27	279 (53.9)	165 (54.1)	114 (53.5)
28–35	171 (33)	102 (33.4)	69 (32.4)
≥ 36	68 (13.1)	30 (12.5)	30 (14.1)
**Accompanied to antenatal care**			
Not accompanied	465 (89.8)	272 (89.2)	193 (90.6)
Accompanied on day of interview	53 (10.2)	33 (10.8)	20 (9.4)
**Use of birth control**			
Did not use	258 (49.8)	163 (53.4)	95 (44.6)
Used 6 months prior to pregnancy	260 (50.2)	142 (46.6)	118 (55.4)
**Type of contraceptive used**			
None or safe days	267 (51.5)	167 (54.8)	100 (47)
Effective contraception	251 (48.5)	138 (45.2)	113 (53)
*HIV-related*			
**HIV status disclosure**			
Disclosed	501 (96.7)	299 (98.0)	202 (94.8)
Not disclosed	17 (3.3)	6 (2.0)	11 (5.2)
**Person disclosed to**			
Husband	398 (76.8)	244 (80.0)	154 (72.3)
Other	120 (23.2)	61 (20.0)	59 (27.7)
**Fear about others' opinion on HIV status**			
Had no fear	253 (48.8)	139 (45.6)	114 (53.5)
Had fear	265 (51.2)	166 (54.4)	99 (46.5)
**Antiretroviral treatment**			
Efavirenz-based	466 (90)	279 (91.5)	187 (87.8)
Nevirapine-based	44 (8.5)	23 (7.5)	21 (9.9)
Protease inhibitor–based	8 (1.5)	3 (0.1)	5 (2.4)
**Duration of antiretroviral treatment**			
Less than 6 months	98 (18.9)	64 (21)	34 (16)
7 to 30 months	117 (22.6)	70 (23)	47 (22)
31 to 119 months	267 (51.5)	159 (52.1)	108 (50.7)
≥ 120 months	36 (7)	12 (3.9)	24 (11.3)

### Predictors for unintended pregnancy

Being single was a significant predictor for unintended pregnancy among HIV infected pregnant women. HIV infected women who were single were almost four times as likely to experience unintended pregnancy as their married counterparts (adjusted odds ratio (AOR) = 3.74, 95% CI: 1.67–8.34). Women who had a higher parity were three times as likely to experience unintended pregnancy as those with lower number or order of pregnancies (parity of ≥ 5; AOR = 2.79, 95% CI: 1.85–4.22). Those who had taken ART for 10 years (120 months) or more were almost four times as likely to report that the pregnancy at the time was unintended as those that had taken ART for less than 6 months (≥ 120 months; AOR = 3.69 (1.57–8.67) (Table 3).

**Table 3:** Predictors for unintended pregnancy among HIV infected women in Northern Uganda.

Variables	Crude OR	*p* value	Adjusted OR	*p* value	Adjusted OR	*p* value
	(95% CI)		(95% CI)^a^		(95% CI)^b^	
**Age**						
≤ 20 years	0.56 (0.27–1.18)	0.13	0.74 (0.3–1.81)	0.505	–	–
21–29 years	0.57 (0.39–0.82)	0.003	0.86 (0.55–1.34)	0.501	–	–
≥ 30 years	1		1		–	–
**Education status**						
0–6 years	1		1		–	–
7–10 years	0.72 (0.49–1.06)	0.098	0.88 (0.57–1.36)	0.563	–	–
11–13 years	0.52 (0.28–0.99)	0.047	0.71 (0.35–1.44)	0.345	–	–
≥ 14 years	0.31 (0.12–0.79)	0.015	0.5 (0.18–1.37)	0.176	–	–
**Marital status**						
Married	1		1		1	
Single	3.75 (1.75–8)	0.01	3.81 (1.68–8.66)	0.001	**3.74 (1.67**–**8.34)**	0.001
**Employment status**						
Employed	1		1		–	–
Not employed	1.27 (0.89–1.81)	0.195	1.13 (0.74–1.73)	0.581	–	–
**Parity**						
≤ 4	1		1		1	
≥ 5	2.83 (1.9–4.2)	0.000	2.4 (1.5–3.86)	0.000	**2.79 (1.85**–**4.22)**	0.000
**HIV status disclosure**						
Disclosed	1		1		1	
Not disclosed	2.71 (0.99–7.46)	0.053	2.55 (0.84–7.76)	0.1	2.59 (0.86–7.78)	0.09
**Socioeconomic status**						
Group 1 (poorest)	1		1		–	–
Group 2	1.04 (0.64–1.70)	0.871	1.25 (0.73–2.14)	0.425	–	–
Group 3	1.05 (0.66–1.64)	0.848	1.44 (0.87–2.38)	0.16	–	–
Group 4 (least poor)	0.61 (0.35–1.07)	0.087	0.91 (0.48–1.73)	0.781	–	–
**Type of contraceptive used**						
None or safe days	1		1		1	
Effective contraception	1.37 (0.96–1.94)	0.081	1.33 (0.9–1.96)	0.148	1.34 (0.92–1.95)	0.132
**Antiretroviral treatment**						
Efavirenz-based	1		1		–	–
Nevirapine-based	1.36 (0.73–2.53)	0.328	0.93 (0.43–1.99)	0.85	–	–
Protease inhibitor-based	2.49 (0.59–10.53)	0.216	2.24 (0.4–12.41)	0.356	–	–
**Duration of antiretroviral treatment**						
0 to 6 months	1		1		1	
7 to 30 months	1.26 (0.72–2.2)	0.409	1.25 (0.68–2.3)	0.482	1.29 (0.71–2.36)	0.398
31 to 119 months	1.28 (0.79–2.07)	0.318	1.15 (0.66–2.01)	0.628	1.19 (0.7–2.03)	0.517
≥ 120	3.76 (1.68–8.45)	0.001	3.32 (1.24–8.92)	0.017	**3.69 (1.57–8.67)**	0.003

The bold figures in Table 3 represent the significant predictors for unintended pregnancy.

^a^R^2^ = 0.0902.

^b^R^2^ = 0.0789.

## Discussion

In this study we sought to determine the prevalence of unintended pregnancy and its predictors among WLH. The predictors for unintended pregnancy in our study were being single, having five or more pregnancies and taking ART for longer durations of time. In this setting we have documented a prevalence of unintended pregnancy of more than 40%. This high rate found in our study is probably due to the unmet need of family planning. Generally studies that report high prevalence of unintended pregnancy also report high rates of unmet need of family planning^6,13,23,24,37,38^ just like in our study. Several studies have reported high prevalence of unintended pregnancy ranging from 35 to 78%^4,7,13,20,23,24,31,38,39^. Studies in Nigeria^4^ and Zimbabwe^20^ reported lower prevalence of unintended pregnancy than our study. This could be attributed to the fact that in both studies, the way the outcome of unintended pregnancy was measured and defined was different from ours. A number of studies did not rely on the definitions of unintended pregnancy stipulated in the LMUP. Studies elsewhere^12,13,22–24,30,31,38,39^ reported higher prevalence of unintended pregnancy than ours. Studies report varying rates because of the difference in the types of population from which they measure unintended pregnancy. Some studies have measured unintended pregnancy among adolescents^39^ which creates a difference in risk factors between these studies and ours. The timing of asking about unintended pregnancy was different between the various studies and our study. Most of the studies asked about intention of the pregnancy long after the women in the cohorts had given birth to their babies not while they were still pregnant like in our study.

In our study, HIV infected women who were single were more likely to experience unintended pregnancy. A plausible explanation for this is that in a marital union there are open communication channels between the two individuals and this facilitates open discussion on reproductive-related issues like child-bearing and contraceptive use. Studies done elsewhere^15,16,31^ also documented that WLH in a relationship were most likely to use contraception if there was open partner discussion among married couples. These communication channels are absent when the woman does not have a partner. Studies in Kenya^39^, Swaziland^22^, Botswana^23^ and South Africa^12,24,31^, demonstrate similar results.

Our study also found that experiencing a higher number of pregnancies increased the likelihood of experiencing unintended pregnancy among WLH. A probable explanation for this finding in our study could be inconsistency in the use of contraception, under-utilisation of emergency contraceptives, side effects of the contraception like heavy bleeding, partner refusal or constant stock outs of contraceptives at health facilities. Our finding was consistent with results from other studies in South Africa^24,31^ and Botswana^23^. However, different results were found in Cameroon^33^ and Uganda^26^. One study in Kenya^39^ showed that the odds of lower order pregnancies to be unintended were the same as those of higher order pregnancies, though the cohort in this study was adolescents which could explain the variation in findings between this study and ours.

It is surprising to note that in our study HIV infected women who had taken ART for long periods of time were more likely to experience unintended pregnancy. Probably, in our study context, women on long-term ART experience complacency and in so doing become non-adherent to both ART and contraceptive use. In a study done in Uganda^40^, it was found that individuals who had taken ART for a long time hardly received continuous ART adherence education, experienced treatment fatigue and were less likely to adhere to their treatment. Studies in South Africa^24,31^ however found that women who had been newly diagnosed with HIV were more likely to experience unintended pregnancy. More qualitative studies need to be done in our study context to understand reasons for this finding.

### Strengths and limitations

The study was conducted in a hospital setting hence our findings may only be generalizable to WLH from within this study context and those similar to it. We did not classify unintended pregnancy into its variations—untimed, unwanted or unplanned. It was difficult to make comparisons between our study and the studies that made these classifications. However, these studies were very few. Women with unintended pregnancy are more likely to seek abortion prior to 20 weeks of gestation. Since these women were not eligible to participate in our study, it is possible that our prevalence estimation was lower than the actual.

Our study had its strengths too. We adapted use of a validated instrument in measuring pregnancy intent. We also measured pregnancy intention with regard to the index pregnancy while the women were pregnant. This discounts the plausibility of recall limitation. However, this does not remove the notion that at the time of measuring pregnancy intent in this cohort, some women might have already come to terms with their pregnancy. We measured pregnancy intent at 20 weeks of pregnancy or more. By this time, a woman might have already accepted and desired an originally undesired pregnancy. We, therefore, could have under reported on the prevalence of unintended pregnancy.

## Conclusions

WLH likely to experience unintended pregnancy included: those who were not in a marital union, those having a higher number of children and those who had taken ART for longer periods. We recommend that alongside integration of family planning services with existing HIV care, counselling services need to target WLH who are not living with a partner, those with high number of children and those that have been in HIV care for long periods of time.

## Data Availability

The datasets used and analysed during the current study are available from the corresponding author on reasonable request.
